# Stability and Thermophysical Properties of GNP-Fe_2_O_3_ Hybrid Nanofluid: Effect of Volume Fraction and Temperature

**DOI:** 10.3390/nano13071238

**Published:** 2023-03-31

**Authors:** Adeola Borode, Thato Tshephe, Peter Olubambi, Mohsen Sharifpur, Josua Meyer

**Affiliations:** 1 Centre for Nanoengineering and Advanced Materials, University of Johannesburg, Johannesburg 2028, South Africa; 2 Department of Mechanical and Aeronautical Engineering, University of Pretoria, Pretoria 0024, South Africa; 3 Department of Medical Research, China Medical University Hospital, China Medical University, Taichung 400, Taiwan; 4 Department of Mechanical and Mechatronic Engineering, Stellenbosch University, Stellenbosch 7600, South Africa

**Keywords:** hybrid nanofluids, graphene nanoplatelets, iron oxide, thermal conductivity, viscosity, heat transfer efficacy

## Abstract

The study focused on the impact of concentration and temperature on the electrical conductivity, viscosity, and thermal conductivity of GNP/Fe_2_O_3_ hybrid nanofluids. The study found that nanofluids have better electrical conductivity, viscosity, and thermal conductivity than water. The electrical conductivity and thermal conductivity increase linearly with concentration for a constant temperature. However, the nanofluid’s viscosity increases with the addition of the hybrid nanoparticles and decreases as the temperature increases. Furthermore, the study shows that the thermal conductivity of the nanofluid is enhanced with increased addition of hybrid nanoparticles in the base fluid and that the thermal conductivity ratio increases with increased addition of nanoparticles. Overall, the results suggest that GNP/Fe_2_O_3_ hybrid nanofluids could be used in various industrial applications to improve the heat transfer and energy efficiency of systems.

## 1. Introduction

Modern machinery and many industrial applications employ traditional cooling fluids such as water, engine oil and glycols for heat transfer applications. The creation of incredibly effective heat transfer devices is greatly influenced by the thermophysical properties of these fluids. However, these fluids exhibit low to modest thermal conductivity (λ), which led to the development of nano-solid-liquid suspensions aimed at improving the λ of common heat transfer fluids. This nano-suspension was first created by Choi et al. in 1995 by adding copper nanoparticles into water [1]. Due to the extraordinary thermal and flow characteristics that the nanofluid exhibits in comparison to those of traditional thermal transportation medium [2,3,4], this unique fluid is the focus of considerable global research. It is projected that nanofluids will be widely used in the future to improve a variety of industrial applications.

Graphene, which is a carbonaceous nanomaterial, is one of the most promising materials for the preparation of nanofluids. This is due to its exceptional thermophysical characteristics, including its great mechanical strength and impressive λ [2,5,6]. A wide variety of other studies [7,8,9] on the thermophysical properties of graphene and other nanomaterials have been conducted. However, in recent years, a new form of nanofluid which comprises two or more different nanomaterials was created in a bid to improve the thermophysical properties and save costs [10,11]. These nanofluids are known as hybrid nanofluids.

Borode et al. [12] studied the thermophysical properties of hybrid GNP/Al_2_O_3_ nanofluid of different particle size ratio in comparison to single GNP nanofluid. The single GNP nanofluid was reported to exhibit a higher λ than the hybrid nanofluids. Notwithstanding, hybridization of the nanofluid was found to reduce the µ of the single GNP nanofluid, which subsequently caused an improvement in the natural convective performance of the nanofluids. The λ and µ of Al_2_O_3_-Cu hybrid nanofluids with different volume contents at room temperature were investigated by Suresh et al [13]. When compared to water, they reported an increase of up to 12.11% and 115% in λ and µ, respectively. Wole-Osho et al. [14] assessed the thermophysical properties of an alumina-ZnO hybrid nanofluid with various mixture ratios for application in a photovoltaic thermal collector. They noticed an increment in the λ and µ with all the addition of the hybrid nanomaterials and with an increase in their nanomaterial loading. Giwa et al. [15] studied the µ and σ of a MWCNT-Fe_2_O_3_ hybrid nanofluid. They observed an enhancement in the properties with an increase in the nanomaterial loading. Adun et al. [16] investigated the effects of temperature, volume concentration, and mixing ratios of alumina-ZnO-Fe3O4 ternary hybrid nanofluids (THNF) on their λ and µ. Three THNF mixture ratios (1:1:1, 1:2:1, and 1:1:2) were synthesized at volume concentrations of 0.5%, 0.75%, 1%, and 1.25%, and all experiments were conducted at temperatures between 25 °C and 65 °C. The results showed that temperature and volume concentration significantly affected the thermophysical properties of the fluid, and the 1:1:1 mixture ratio had the highest λ enhancement of 36.018%. Additionally, the 1:1:1 mixture ratio had the lowest viscosity, while the 2:1:1 THNF mixture ratio had the highest viscosity.

The literature and a host of other studies [17,18,19,20] provide evidence that the thermophysical properties of base fluids will improve with the application of hybrid nanoparticles. Thus, the thermophysical properties of graphene-based nanofluids can be further enhanced through hybridization. However, as far as the authors are aware, little to no study has been completed on the graphene-based ferrofluid.

This study aims to investigate the effect of concentration and temperature on the thermophysical properties of a novel hybrid GNP/Fe_2_O_3_ nanofluid with a mixing ratio of 50:50. To the best of the authors’ knowledge, no study has been completed using hybrid GNP/Fe_2_O_3_ nanomaterial. The choice of GNP/Fe_2_O_3_ in the formulation of a hybrid nanofluid is based on the desired properties of the resulting fluid. GNPs have high thermal conductivity and stability, making them attractive as heat transfer enhancers in nanofluids [2]. On the other hand, Fe_2_O_3_ nanoparticles are readily available and cost-effective compared to other types of nanoparticles such as metals or carbon nanotubes [21]. This makes them an attractive choice in the development of efficient and affordable nanofluids. Additionally, Fe_2_O_3_ nanoparticles are magnetic [22], allowing for easy manipulation and separation of the nanofluid using an external magnetic field. Fe_2_O_3_ nanoparticles are also known to produce stable water-based nanofluids, which have been extensively studied in the literature [23,24,25]. This suggests that incorporating Fe_2_O_3_ nanoparticles into a hybrid nanofluid could improve its stability and overall performance. Therefore, the combination of GNP and Fe_2_O_3_ in a hybrid nanofluid can potentially enhance its thermal conductivity and convective heat transfer while allowing for easy separation and reusability. The stability of the nanofluids was evaluated using a visual observation approach and TEM analysis. The study was performed for volume concentrations ranging from 0.1 vol% to 0.4 vol%. The temperature ranged between 15 °C and 40 °C for the thermal conductivity λ measurement, while it ranged between 15 °C and 55 °C for the µ and electrical conductivity (σ) measurements. Finally, a novel correlation was developed using regression analysis to estimate the measured thermophysical characteristics of the hybrid nanofluid as a function of temperature and concentration.

## 2. Materials and Methods

In this study, deionized water was used as the base fluid due to its higher λ_water_ and lower µ_water_ compared to other base fluids such as ethylene glycol. The hybrid nanofluid was prepared using a mixture of GNP and nanomagnetic Fe_2_O_3_ with a weight ratio of 50:50. The GNP nanomaterial with thickness of 15 nm and specific surface area of 50–80 m^2^/g was acquired from Sigma Aldrich (DE), while the Fe_2_O_3_ with length of 10–30 µm and external diameter of 10–20 nm was obtained from MKnano Company (CA). The hybrid nanofluids were stabilized using SDS surfactant obtained from Sigma Aldrich (DE) at a nanoparticle/surfactant ratio of 1:1. Equation (1) was used to compute the weight of the nanomaterials (φ).

(1) $$\varphi =\frac{\omega_{GNP}{\left(\frac{m}{\rho }\right)}_{GNP}+\omega_{Fe_{2}O_{3}}{\left(\frac{m}{\rho }\right)}_{Fe_{2}O_{3}}\;}{\omega_{GNP}{\left(\frac{m}{\rho }\right)}_{GNP}+\omega_{Fe_{2}O_{3}}{\left(\frac{m}{\rho }\right)}_{Fe_{2}O_{3}}+{\left(\frac{m}{\rho }\right)}_{water}\;}$$

where ω,  m, ρ is the weight fraction, mass and density of the nanoparticle, respectively.

**Figure 1:**
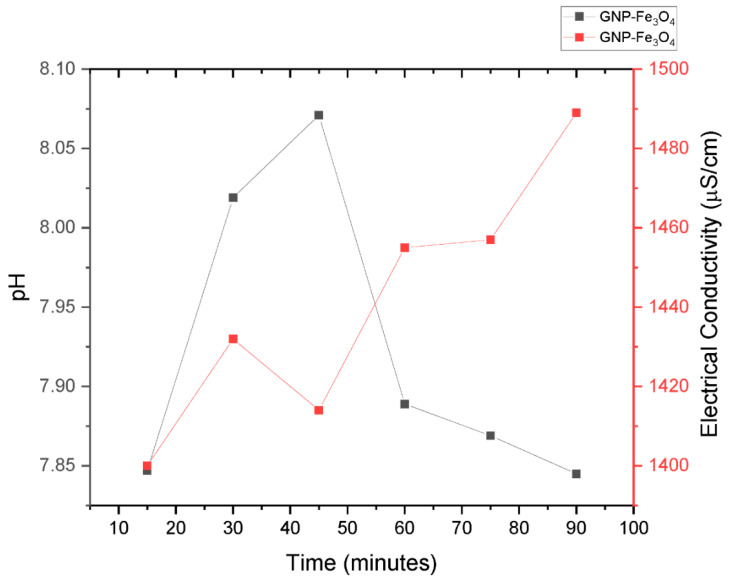
pH and electrical conductivity as a function of ultrasonication time.

The hybrid nanofluids were prepared by first dispersing the nanoparticles and surfactant in deionized water using a Q-700 Qsonica ultrasonic agitator for an optimum time to ensure proper dispersion. During the agitation process, the nanofluid was placed in a water bath (LAUDA ECO) with a set temperature of 20 °C to avoid overheating and evaporation. Further, the stability of the nanofluid was monitored using visual observation technique and TEM. To achieve stable hybrid nanofluids, the sonication time needed to be optimized. The pH and σ are useful properties for determining the critical micelle concentration (CMC) of the surfactant used in nanofluid preparation [15]. The CMC is the point at which surfactant molecules form micelles that can stabilize nanoparticles in the nanofluid [26]. The CMC is identified as the inflection point in the pH and σ curve for varying sonication time. In this study, the optimum sonication time was determined by monitoring the pH and σ of the hybrid nanofluid for different sonication times at an ambient temperature as shown in Figure 1. A point of inflection was observed for the two properties at 45 min to indicate the optimum sonication time used to prepare all the hybrid nanofluid used in this study.

The hybrid nanofluids were characterized by measuring their µ, λ and σ using a Vibro-viscometer (SV-10), KD-2 Pro meter, and EUTECH electrical conductivity meter (CON700), respectively. The µ and σ were measured for a temperature range between 15 °C and 55 °C, and a water bath (LAUDA ECO) was used to regulate the temperature. Additionally, the λ was measured at a temperature range between 15 °C and 40 °C.

The electrical conductivity meter was calibrated using the calibration fluid provided by the manufacturer, and the standard fluid was measured three times at room temperature to obtain an average value of 1414 µS/m, which was found to be close to the manufacturer’s specified value of 1413 µS/m. The reliability of both the viscometer and KD2Pro Meter was assessed by measuring their µ and λ and comparing them to the established standard values for water found the literature.

To ensure accurate measurement of the thermophysical properties (*P*), potential sources of error were identified, including the measurement of the weight of nanomaterials and surfactants (*W*), volume of water (*V*), and temperature (*T*). These errors were accounted for by using Equation (2) to estimate the uncertainty (*e*) associated with the properties.

(2) $$e\;\left(\%\right)=\pm \sqrt{{\left(\frac{\partial P}{P}\right)}^{2}+{\left(\frac{\partial W}{W}\right)}^{2}+{\left(\frac{\partial V}{V}\right)}^{2}+{\left(\frac{\partial T}{T}\right)}^{2}}$$

The degree of uncertainty associated with the measurement of σ is ±2.06%, while for λ and µ, it is ±2.12% and ±2.07%, respectively.

## 3. Results and Discussion

### 3.1. Nanofluid Stability

**Figure 2:**
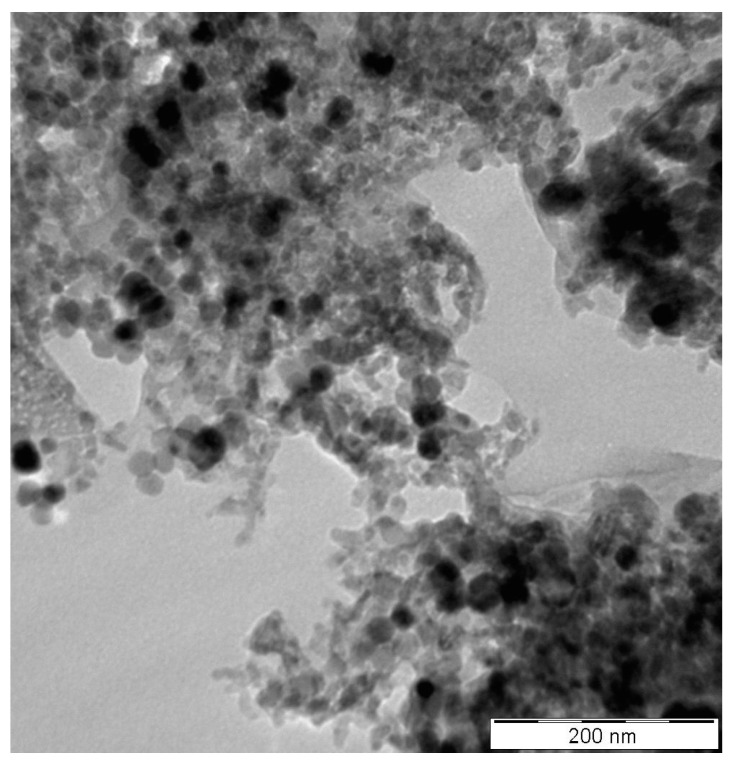
TEM Images of the GNP/Fe_2_O_3_ hybrid nanofluid.

**Figure 3:**
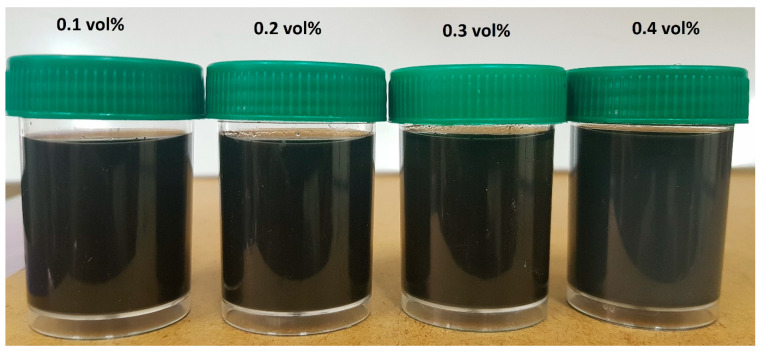
Photographs of the hybrid nanofluids 4 days after preparation.

**Figure 4:**
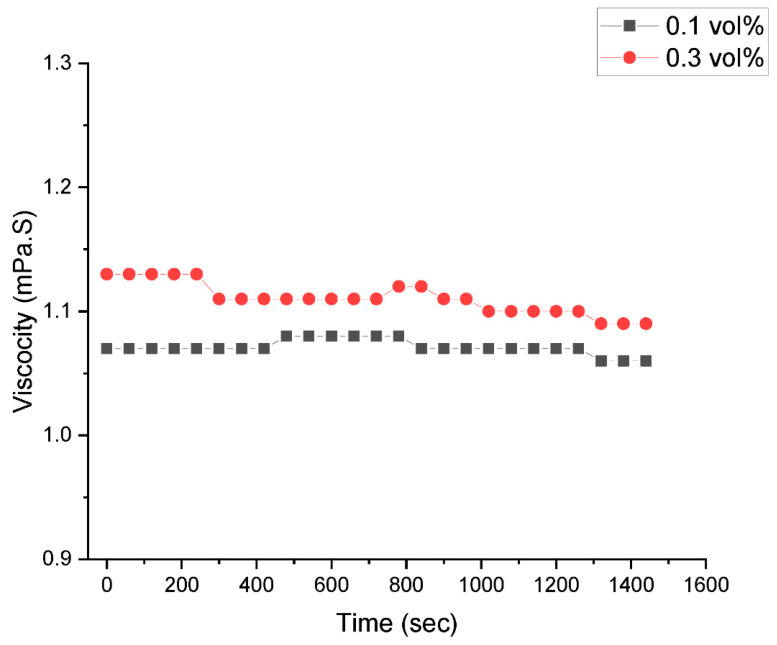
Viscosity-based stability measurement for 0.1 vol% and 0.3 vol% hybrid nanofluids.

Figure 2 displays the TEM micrograph of the GNP/Fe_2_O_3_ hybrid nanofluid. The homogenous dispersion of the GNP and Fe_2_O_3_ nanomaterials is clearly evident in the micrograph. To demonstrate the nanofluids’ improved stability, other stability evaluation techniques, including visual and µ measurement techniques, were employed. The photographic image of the nanofluids after 4 days is presented in Figure 3. To validate the stability, the **µ_HNF_** was also monitored for 24 h, as illustrated in Figure 4. It is evident that the nanofluid with 0.1 vol% and 0.3 vol% maintains average stability over a period of 24 h due to the negligible changes in the **µ_HNF_** over the examined duration.

### 3.2. Viscosity

**Figure 5:**
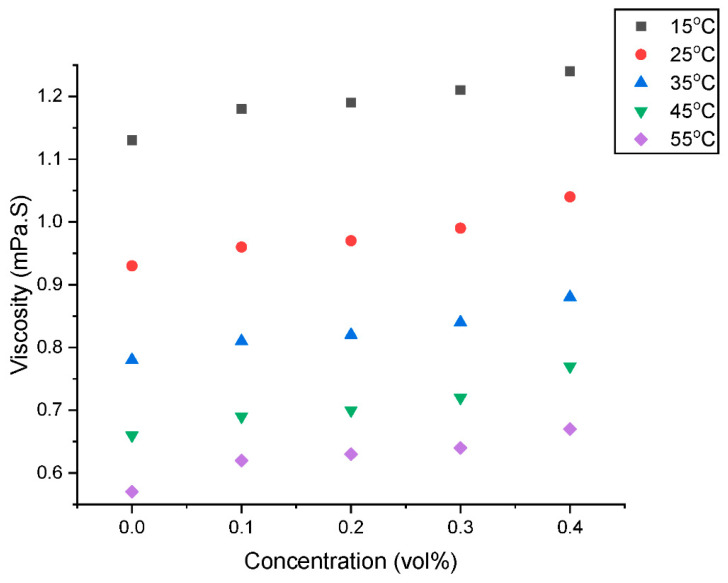
Influence of concentration on the viscosity of the hybrid nanofluids at various temperatures.

A fluid’s dynamic viscosity is the key component that determines how it behaves and travels in close proximity to solid boundaries. Additionally, it demonstrates a considerable influence on the pressure drop and pumping efficiency in any industrial system. Figure 5 shows the results of a measurement of the viscosity (µ_HNF_) of a GNP/Fe_2_O_3_ hybrid nanofluid at volume concentration of 0.1–0.4% and temperatures of 15–40 °C. Considering the changes in µ_HNF_ at various volume concentrations, it can be seen that adding GNP/Fe_2_O_3_ nanoparticles at a steady temperature increases the µ_HNF_.

**Figure 6:**
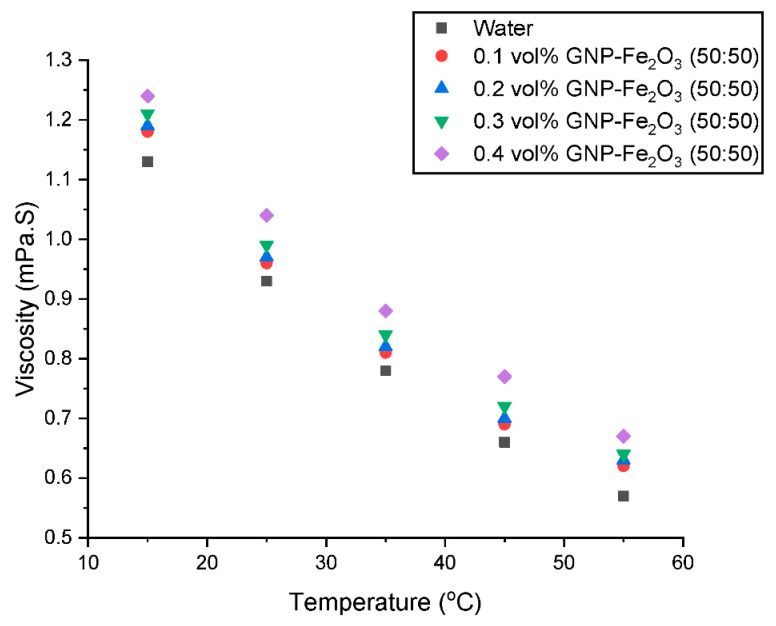
Influence of temperature on the viscosity of the hybrid nanofluids at various concentrations.

Whilst also analyzing the changes in µ_HNF_ at various temperatures as shown in Figure 6, it is easy to see that, for a given volume fraction, the µ_HNF_ declines as temperature rises. The increase in temperature accelerates the Brownian motion of fluid molecules, which is what causes the µ_HNF_ to lower. By increasing the frequency of molecular collisions as the velocity increases and decreasing the intermolecular interactions, µ_HNF_ lowers. Because temperature enhances the Brownian motion, there is an inverse relationship between temperature and µ_HNF_.

The µ_HNF_ is decreased as a result of the lessening of intermolecular forces. In other words, µ_HNF_ decreases as temperature increases. Another explanation is that when the temperature rises, the distance between molecules in the base fluid and the nanoparticles shortens, thus, reducing µ_HNF_ and flow resistance. This study outcome is well-supported and validated by the existing literature [12,27,28].

**Figure 7:**
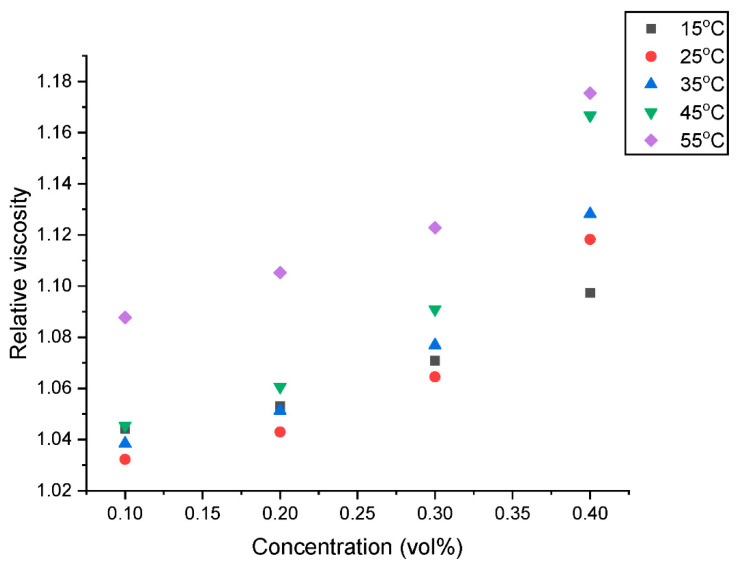
Influence of concentration on the relative viscosity of the hybrid nanofluids at various temperatures.

**Figure 8:**
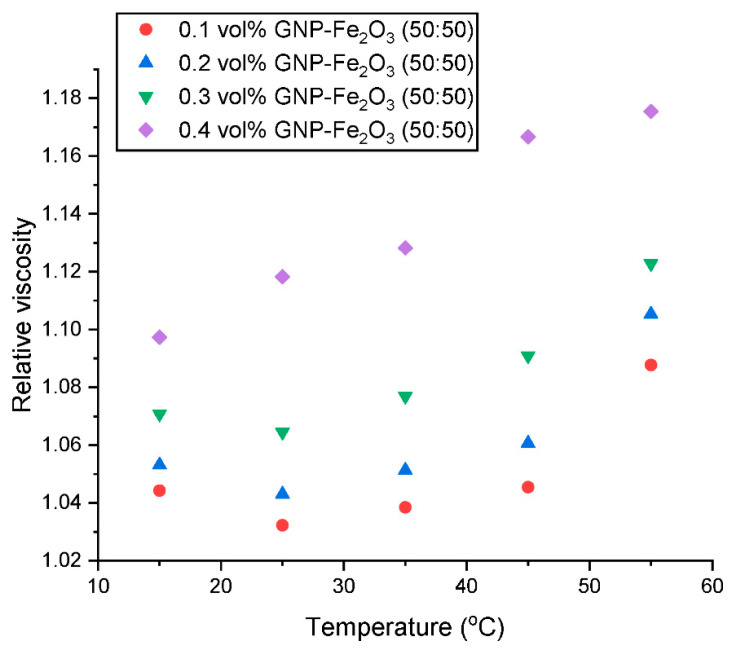
Influence of temperature on the relative viscosity of the hybrid nanofluids at various concentrations.

The relative dynamic viscosity (µ_relative_) is presented in Figure 7 and Figure 8 as a function of concentration and temperature, respectively. Figure 7 shows that there is an augmentation in the µ_relative_ with an increased addition of hybrid nanoparticles in the nanofluids. This observation is well-supported by the existing literature [15,16,21]. This increase in µ_relative_ could be attributed to the build-up of some agglomerates with a rising concentration within the nanofluid. Figure 8 shows a slight diminution in µ_relative_ with an increase in temperature from 15 °C to 20 °C. However, an increase in temperature above 20 °C results in an increase in the µ_relative_. According to this study, a µ increment of 3.22–8.77%, 4.30–10.53%, 6.45–12.28% and 8.60–15.79% was observed for the hybrid nanofluid with GNP/Fe_2_O_3_ loading of 0.10, 0.20, 0.30 and 0.40 vol% respectively at 15–40 °C.

### 3.3. Thermal Conductivity

**Figure 9:**
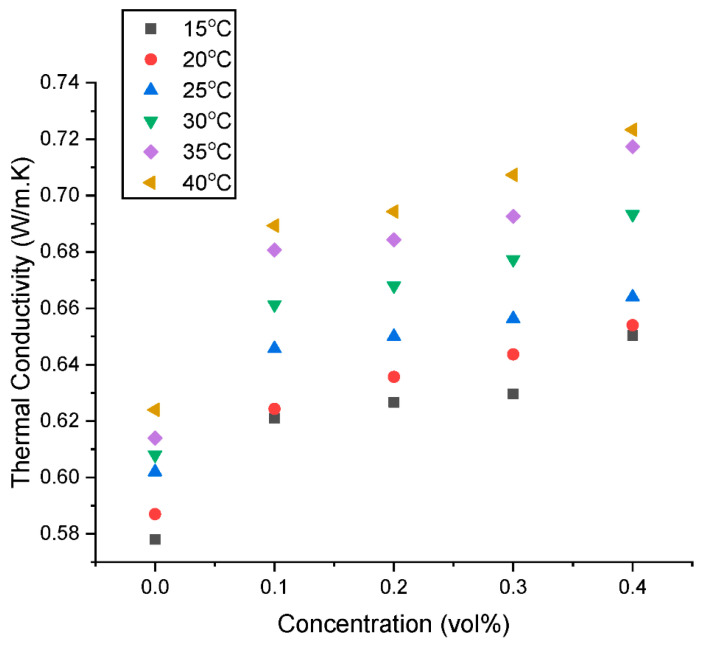
Influence of concentration on the thermal conductivity of the hybrid nanofluids at various temperatures.

**Figure 10:**
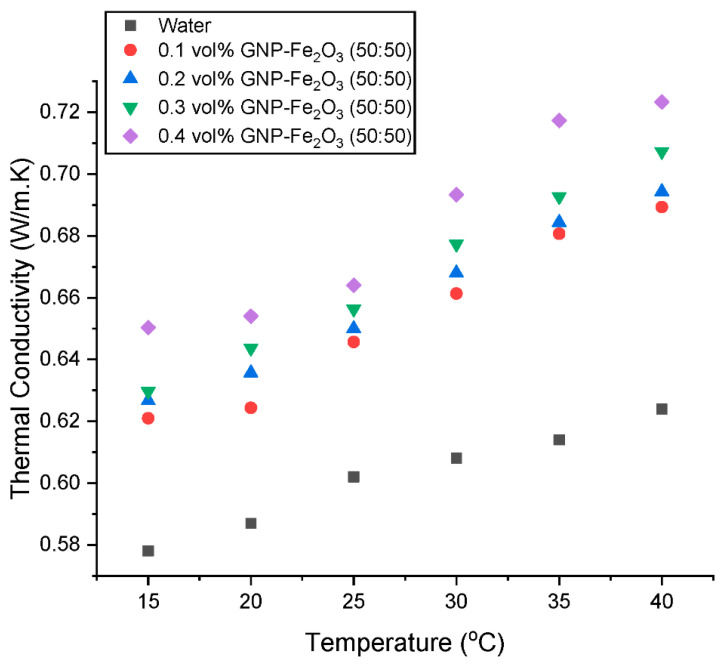
Influence of temperature on the thermal conductivity of the hybrid nanofluids at various concentrations.

**Figure 11:**
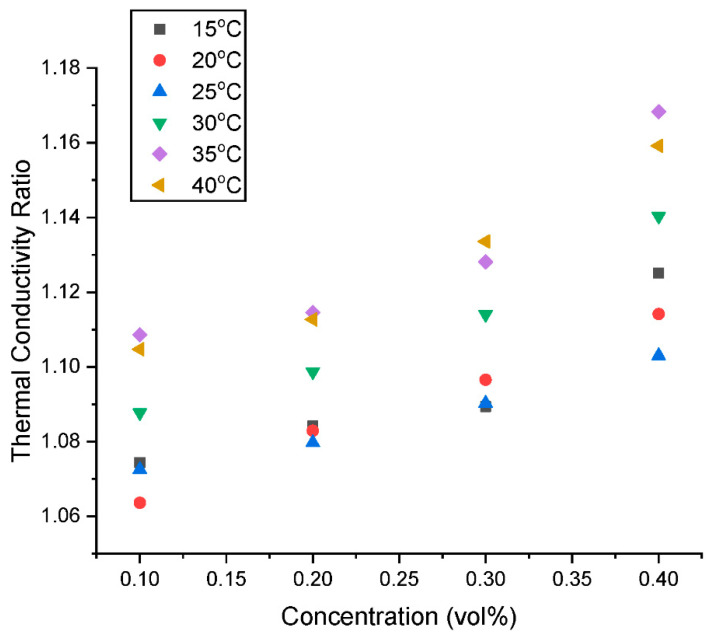
Influence of concentration on the thermal conductivity ratio of the hybrid nanofluids at various temperatures.

**Figure 12:**
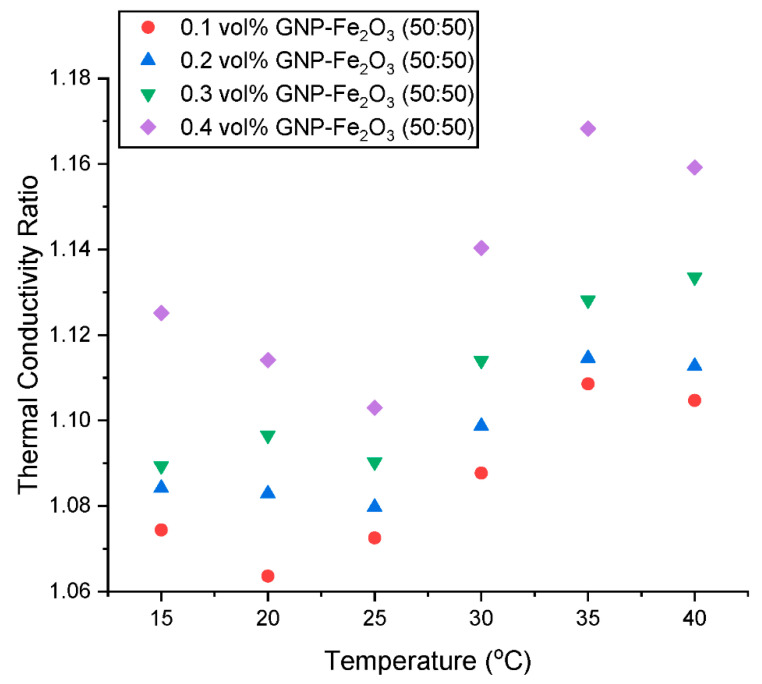
Influence of temperature on the thermal conductivity ratio of the hybrid nanofluids at various concentrations.

The thermal conductivity (λ_HNF_) of a hybrid GNP/Fe_2_O_3_ nanofluid at volume fractions of 0.1–0.4% was assessed at temperature of 15–40 °C. The outcomes are shown in Figure 9, Figure 10, Figure 11 and Figure 12. Figure 9 shows that at steady temperature the λ_HNF_ is increased in comparison to the base fluid by the addition of the hybrid nanoparticles in the base fluid. Additionally, as the volume percentage of nanoparticles grows, the surface-to-volume ratio rises. This process ultimately causes the λ_HNF_ coefficient to rise significantly.

Additionally, when the volume fraction grows, there are more intermolecular collisions between molecules, which increases the amount of temperature effects on the λ_HNF_ coefficient. The effect of temperature is further illustrated in Figure 10, as the λ_HNF_ is augmented at a higher temperature. The primary causes of the increase in fluid λ_HNF_ with temperature are Brownian motion and collisions between nanoparticles [29]. This augmentation can also be attributed to improved heat transfer at particle-fluid interface.

The thermal conductivity ratio (λ_ratio_) is presented in Figure 11 and Figure 12 as a function of concentration and temperature, respectively. Figure 11 shows that there is an augmentation in the λ_ratio_ with an increased addition of hybrid nanoparticles in the nanofluids. This increase could be attributed to the build-up of some agglomerates with a rising concentration within the nanofluid. Figure 12 shows a slight diminution in λ_ratio_ with an increase in temperature from 15 °C to 20 °C. However, an increase in temperature from 20 °C to 30 °C results in an increase in the λ_ratio_ before it declines as the temperature is increased to 40 °C. The λ_ratio_ is influenced by the intermolecular forces between the nanoparticles and the base fluid. When the temperature is raised from 15 °C to 20 °C, the intermolecular forces between the nanoparticles and the base fluid start to weaken due to thermal motion and vibrations of the particles, which causes a reduction in λ_ratio_. However, as the temperature is further elevated from 20 °C to 25 °C, the Brownian motion of the nanoparticles becomes more significant, leading to better dispersion and contact between the nanomaterial and the base fluid. As a result, the intermolecular forces increase, leading to an increase in λ_ratio_. At higher temperatures above 30 °C, the λ_ratio_ starts to decrease again due to the formation of agglomerates and the decrease in intermolecular forces caused by the increased thermal motion of the particles. This behavior is commonly observed in nanofluids and is attributed to the complex interplay between the thermal energy, Brownian motion, and intermolecular forces between the nanoparticles and the base fluid. It is important to note that the exact reasons for the observed trends in λ_ratio_ may depend on several factors, such as the type and size of the nanoparticles and the experimental conditions. Therefore, further research may be needed to fully understand the underlying mechanisms.

According to this study, a thermal conductivity augmentation of 6.36–10.85%, 8.29–11.45%, 8.94–13.35% and 10.30–16.83% was observed for the hybrid nanofluid with a nanoparticle loading of 0.10, 0.20, 0.30, and 0.40 vol% respectively at 15–40 °C. The λ_HNF_ results and trends of this study are well supported and validated by existing studies [12,30,31].

### 3.4. Electrical Conductivity

**Figure 13:**
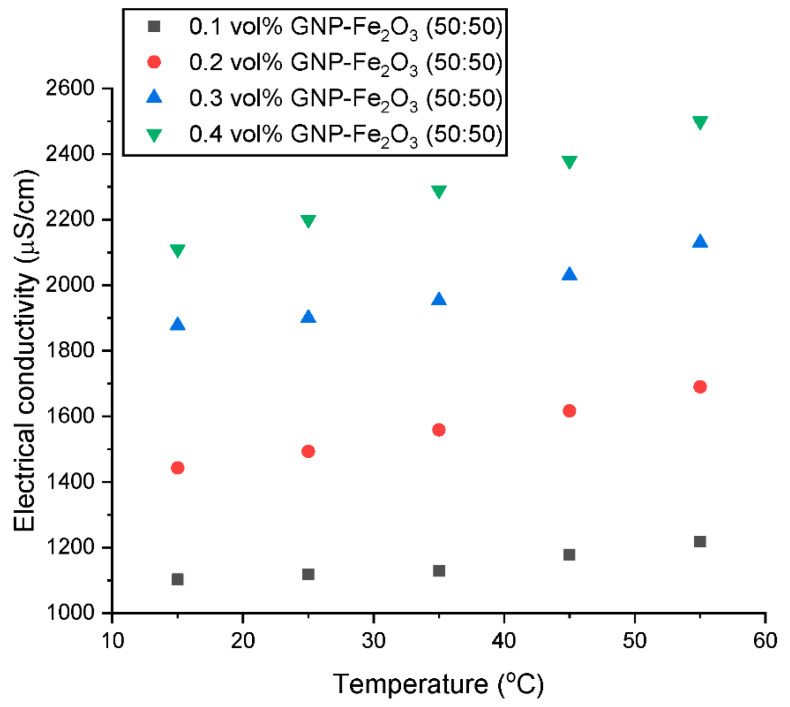
Influence of temperature on the electrical conductivity of the hybrid nanofluids at various concentrations.

**Figure 14:**
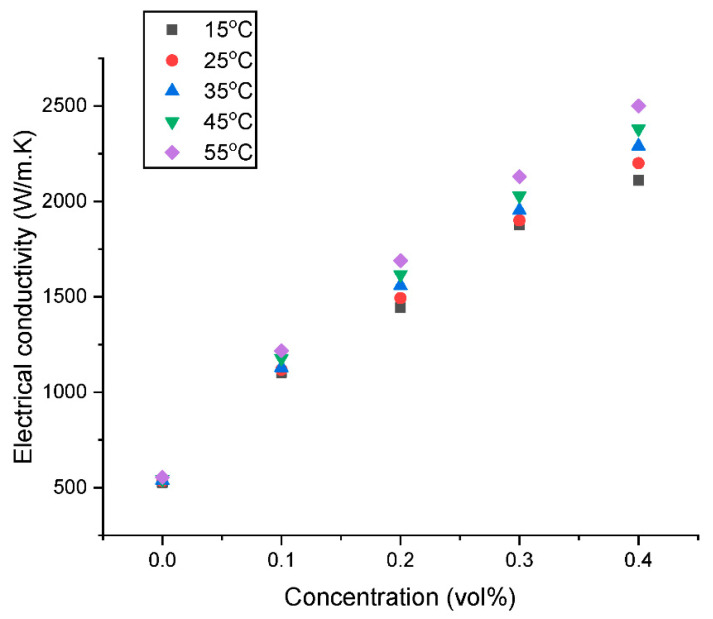
Influence of concentration on the electrical conductivity of the hybrid nanofluids at various temperatures.

Although it has not received much attention, σ is a crucial feature for the technical application of nanofluids. Figure 13 and Figure 14 depict the effect of concentration and temperature, respectively on the electrical conductivity of the GNP/Fe_2_O_3_ hybrid nanofluid (σ_HNF_). The figures show that, when compared to water, all nanofluids have a better σ_HNF_ than water. Similar to the λ_HNF_ observation, the σ_HNF_ increases linearly with concentration for a constant temperature. The improvement in σ_HNF_ is facilitated by the formation of the electrical double layer (EDL) on the surface of the hybrid nanoparticle when dispersed in deionized water. The formation of the EDL is impacted by the polarity of water, which advances the growth of charges on the hybrid nanofluid’s surface and subsequently transfers charges into the nanofluid solution.

As shown in Figure 14, the σ_HNF_ of the GNP/Fe_2_O_3_ hybrid nanofluid also rises with an increase in the nanoparticle loading, following a similar pattern to that of the thermal conductivity. The temperature-induced augmentation could be ascribed to the fact that an elevated temperature makes ions more mobile, subsequently augmenting the σ_HNF_. This improved ion mobility can be attributed to the Brownian motion of the nanomaterials in the base fluid, which can enhance the contact between particles and result in more efficient electrical conduction paths. This increased Brownian motion can also disrupt the electric double layer surrounding the particles, resulting in a decrease in the electrical resistance and an increase in the σ_HNF_. In addition, the temperature elevation results in a reduction in the µ_HNF_, which subsequently improves the mobility of the nanoparticles and promotes their suspension stability in the fluid. This can facilitate the formation of conductive pathways between the particles, leading to an increase in σ_HNF_. The increase in σ_HNF_ with increasing nanoparticle loading and temperature is consistent with previous studies [7,12,30] on the electrical properties of nanofluids.

**Figure 15:**
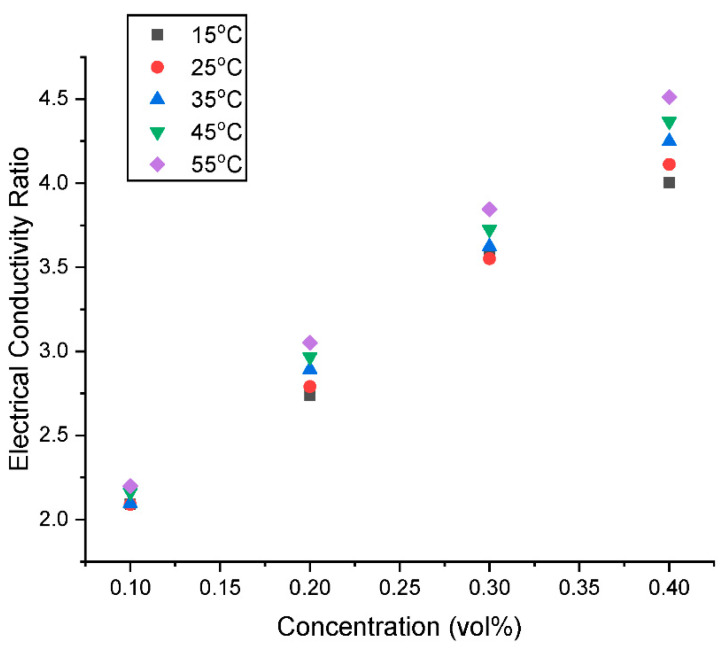
Influence of concentration on the electrical conductivity ratio of the hybrid nanofluids at various temperatures.

**Figure 16:**
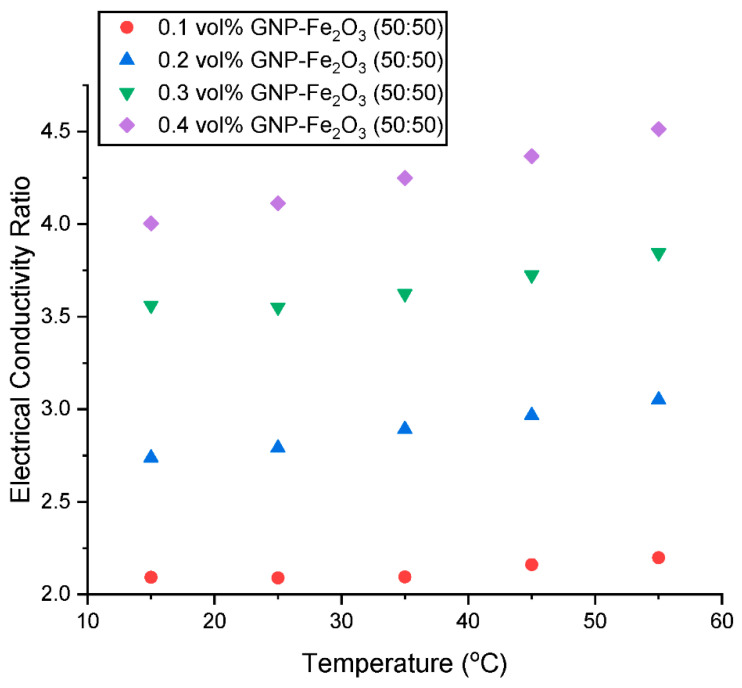
Influence of temperature on the electrical conductivity ratio of the hybrid nanofluids at various concentrations.

The σ_ratio_ is presented in Figure 15 and Figure 16 as a function of concentration and temperature, respectively. It can be observed that there is a linear increase in σ_ratio_ with an increase in concentration and temperature. At constant temperature of 15 °C, the σ_water_ is augmented by 108.97% with the addition of 0.1 vol% GNP/Fe_2_O_3_ nanoparticle up to 300.38% for 0.4 vol%. Moreover, with a nanomaterial loading of 0.1 vol%, the σ_HNF_ is increased from enhancement of 108.97% at 15 °C up to 119.86% at 55 °C. The maximum enhancement was observed to be 351.26% for 0.4 vol% hybrid nanofluid at 55 °C.

### 3.5. Heat Transfer Efficacy

To evaluate the cooling effectiveness or heat transfer efficacy of the hybrid nanofluid, the idea of properties enhancement ratio (PER) is utilized [32]. The *PER* of nanofluids is typically calculated based on the *λ*_HNF_ and µ_HNF_, which are key parameters governing the heat transfer performance of the fluid. The PER can be calculated using Equation (1) [33].

(3) $$PER=\frac{\left(\mu_{relative}-1\right)}{\left(\lambda_{ratio}-1\right)}$$

It is important to note that hybrid nanofluids have high potential for heat transfer when the *PER* value is below 5, while a nanofluid with a *PER* value higher than 5 demonstrates poor thermal performance [32]. Thus, a lower *PER* value indicates better heat transfer performance for nanofluids as a cooling medium.

**Figure 17:**
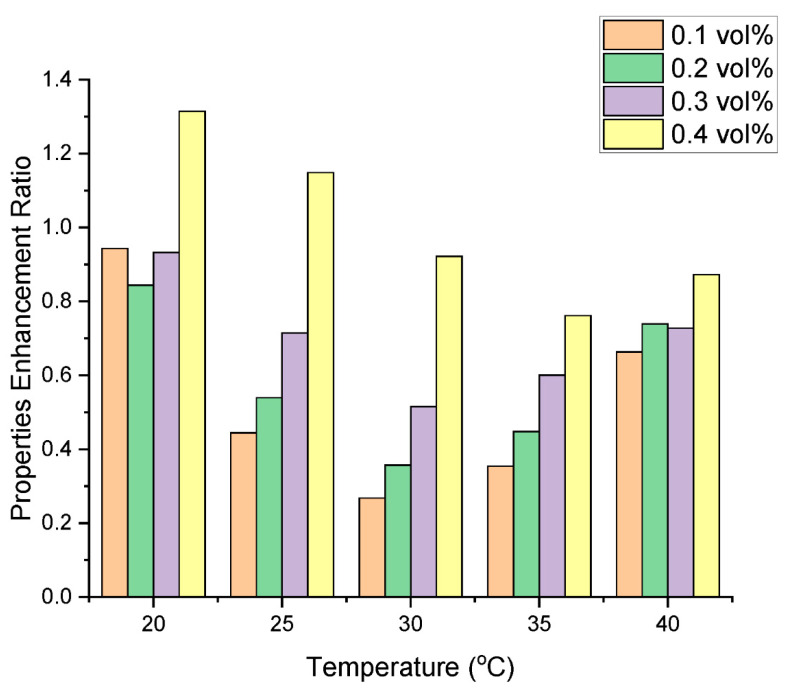
PER values of the hybrid nanofluid in relation to temperature and concentration.

In Figure 17, the *PER* of the hybrid nanofluids is illustrated as a function of volume fraction and temperature. The results indicate that all the prepared hybrid nanofluids are suitable as coolants, as their *PER* values are much lower than 5, with the highest value of 1.31 obtained for a GNP/Fe_2_O_3_ loading of 0.4 at 20 °C. This shows that an increase in the concentration of nanoparticles raises the *PER* value. However, there is a reduction in the *PER* value when the temperature is elevated. This could be attributed to the temperature-induced reduction in µ_HNF_ and augmentation in λ_HNF_ due to Brownian motion. The low *PER* values observed in this study indicate that GNP/Fe_2_O_3_ hybrid nanofluids are favrable for high temperature applications.

### 3.6. Correlation

Various models have been used to predict the thermophysical properties of nanofluids, but classical models may not be suitable for predicting the properties of advanced nanofluids such as hybrid nanofluids [15,34,35,36]. Thus, new models are needed to accurately predict the properties of these advanced nanofluids. In this study, the authors used experimental data to develop prediction models for the thermophysical properties of the hybrid nanofluids. Regression analysis was used to create correlations between volume fraction, temperature, and the experimental data (λ_ratio_, µ_relative_ and σ_ratio_) of the nanofluids, allowing for predictions of these properties.

**Figure 18:**
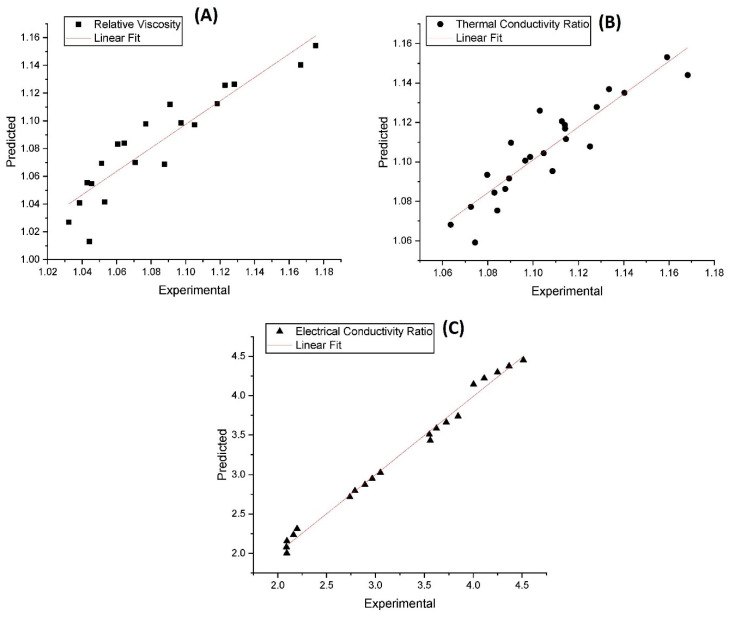
Linear fitting of the experimental and predicted values of the thermophysical properties. (**A**) Relative viscosity; (**B**) Thermal Conductivity Ratio; (**C**) Electrical Conductivity Ratio.

Table 1 shows the correlation equation, Pearson correlation coefficient (R), the correlation of determination (R^2^), and the root-mean-square error (RSME) developed for the temperature-dependent λ_ratio_, µ_relative,_ and σ_ratio_ of GNP-Fe_2_O_3_ hybrid nanofluids at different nanomaterial loading. All the correlations demonstrated high correlation coefficients, with considerably low errors, as indicated by these variables in Table 1. The linear fitting of the experimental and predicted values of the thermophysical properties are shown in Figure 18A–C. The figures indicate that there is a strong correlation between the predicted data and the experimental data for all the properties with minimal deviations as observed in Figure 18A,B. It was noticed that the established relationship between μ_HNF_ and λ_HNF_ was able to forecast the experimental results with a margin of deviation (MOD) within a range from −2.14 to 2.99% and −2.08 to 2.07%, correspondingly. Similarly, the range of difference between the predicted and experimental values of σ_HNF_ was found to be between −5.14 and 4.35%.

## 4. Conclusions

This study investigated the impacts of concentration and temperature on the σ_HNF_, µ_HNF_, and λ_HNF_ of GNP/Fe_2_O_3_ hybrid nanofluids. The findings showed that the σ_HNF_, λ_HNF_, and µ_HNF_ were notably increased in comparison to the base fluid. The boost in electrical conductivity was due to the creation of an electrical double layer (EDL) on the surface of the hybrid nanoparticles, which was influenced by the polarity of water. The λ_HNF_ increase was ascribed to the increase in the surface-to-volume ratio and the number of intermolecular collisions between nanoparticles. Brownian motion and collisions between nanoparticles were identified as one of the primary causes of the increase in λ_HNF_ with temperature.

Furthermore, the µ_HNF_ was found to increase with the addition of nanoparticles at a steady temperature, while it decreased with an increase in temperature due to the lessening of intermolecular forces. The increase in µ_relative_ with an increase in concentration was attributed to the formation of some agglomerates within the nanofluid. In addition, the regression formula developed to establish the relationship between σ_HNF_, λ_HNF_, and µ_HNF_ as a function of temperature and concentration corresponds well with the experimental data.

Overall, the results suggest that GNP/Fe_2_O_3_ hybrid nanofluids have excellent thermal and electrical conductivity properties, which make them suitable for various industrial applications such as heat transfer fluids, lubricants, and coolants. However, the study also highlights the importance of careful consideration of the concentration and temperature effects on the properties of nanofluids, as they can significantly impact the performance of the fluid in various applications.

## Figures and Tables

**Table 1:** Correlation equation for the thermophysical properties of the GNP-Fe_2_O_3_ hybrid nanofluid.

Property	Equation	R	R^2^	RSME
µ_relative_	$\frac{\mu_{HNF}}{\mu_{Water}}=0.96360+0.2852\varphi +0.001393T$	0.91999	0.8464	0.015968
λ_ratio_	$\frac{\lambda_{HNF}}{\lambda_{Water}}=1.01568+0.1625\varphi +0.001811T$	0.91291	0.8334	0.010666
σ_ratio_	$\frac{\sigma_{HNF}}{\sigma_{Water}}=1.17200+7.1380\varphi +0.007740T$	0.99596	0.9919	0.072709

## Data Availability

The data presented in this study are available in the article.
